# Combined Endocardial Pulsed Field: Radiofrequency Ablation of a 3-Dimensional Ventricular Tachycardia With MidMyocardial Isthmus

**DOI:** 10.1016/j.jaccas.2026.109564

**Published:** 2026-07-31

**Authors:** Mayara Marques, Maxime Cerantola, Alexios Hadjis

**Affiliations:** Division of Electrophysiology, Department of Medicine, Hôpital du Sacré-Coeur de Montréal, Université de Montréal, Montreal, Quebec, Canada

**Keywords:** epicardial, pulsed field ablation, 3-dimensional mapping, ventricular tachycardia

## Abstract

**Background:**

Epicardial access for ventricular tachycardia (VT) ablation may be limited in patients with previous cardiac surgery. Substrate depth is a key determinant of ablation success, and deep intramural circuits may not be adequately targeted with conventional endocardial radiofrequency (RF) ablation alone.

**Case Summary:**

Recurrent VT in a patient with previous cardiac bypass surgery and failed endocardial RF ablation. Mapping demonstrated a midmyocardial isthmus, successfully targeted with VT interruption by using focal combined endocardial pulsed field (PF) and RF ablation.

**Discussion:**

Emerging in vivo evidence suggests that successive PF applications, as well as combined RF-PF ablation, can enhance lesion depth. This case demonstrates immediate VT termination and subsequent noninducibility with successive endocardial PF-RF lesions directly apposed to the far-field midmyocardial/epicardial isthmus, demonstrating the critical depth achieved in real time.

**Take-Home Message:**

Hybrid RF-PF energy delivery may expand the therapeutic options for complex multiplanar VT circuits when epicardial access is not feasible.

**Visual Summary:**
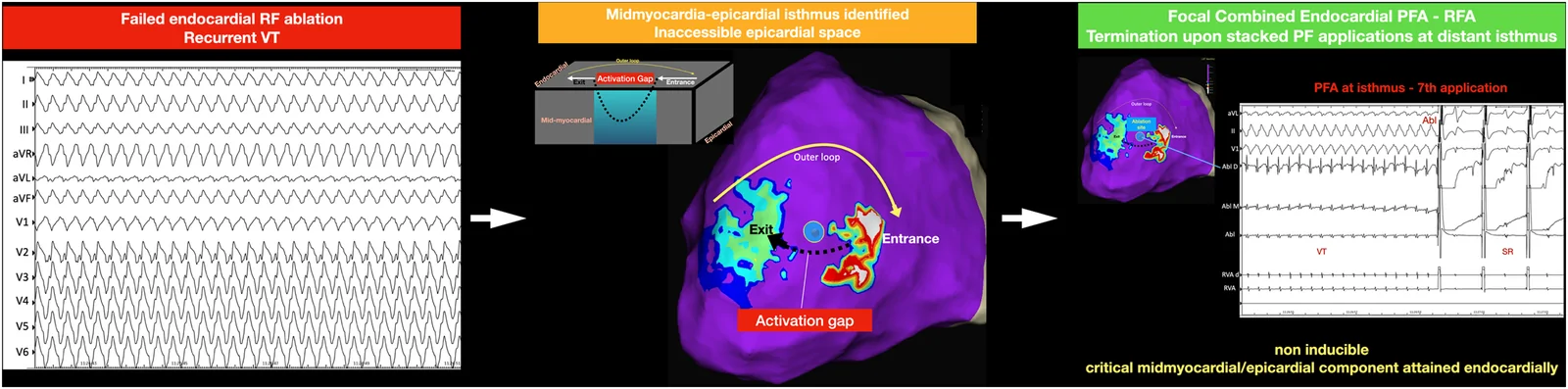
Immediate VT Termination With Successive Endocardial PF-RF Lesions Directly Apposed to Far-Field Midmyocardial Isthmus (Left) 12-lead electrocardiogram of the recurrent clinical VT. (Middle) Graphical representation of the multiplanar nature of the VT circuit alongside the VT activation map with midmyocardial/epicardial isthmus. The wave of propagation progresses from the entrance on the endocardium, then disappears beneath the surface, and finally re-emerges at the exit. (Right) Ablation catheter positioned endocardially atop the suspected midmyocardial/epicardial isthmus. VT termination occurred upon the seventh PF application, once critical ablation depth was achieved with successive applications at the same site. Abl = ablation; Abl D = ablation catheter distal; Abl M = ablation catheter mid; LAT = local activation time; PF = pulsed field; PFA = pulsed field ablation; RF = radiofrequency; RFA = radiofrequency ablation; RVA = right ventricular apex proximal; RVA d = right ventricular apex distal; SR = sinus rhythm; VT = ventricular tachycardia.

Epicardial ablation of ventricular tachycardia (VT) is particularly challenging in patients with ischemic cardiomyopathy who have undergone coronary artery bypass grafting and have a suspected transmural substrate. Pericardial adhesions and the risk of injury to bypass grafts may limit or preclude epicardial access^1^ despite an arrhythmogenic substrate that frequently extends to the epicardium.^2^ In this context, achieving sufficient lesion depth from an endocardial approach becomes critical to effectively target intramural and epicardial circuits.

**Take-Home Messages**

• Substrate depth is a key determinant of ablation success, and deep intramural or epicardial circuits may not be adequately targeted with conventional endocardial radiofrequency ablation alone.
• Hybrid radiofrequency–pulsed field energy delivery may expand the therapeutic options for complex multiplanar ventricular tachycardia circuits when epicardial access is not feasible.

Radiofrequency (RF) ablation, the cornerstone of VT ablation, may be limited by inadequate lesion penetration in deep myocardial substrates. Pulsed field ablation (PFA) has recently emerged as a nonthermal modality that induces myocardial injury through electroporation and may complement conventional RF ablation.

Experimental data suggest that colocalized RF–pulsed field (PF) lesion “stacking” increased lesion depth and width compared with either modality alone, supporting its use when targeting suspected midmyocardial/epicardial substrates.^3^

We describe a case of a 3-dimensional mapped VT circuit successfully eliminated with endocardial ablation using a focal combined PF and RF ablation catheter.

## History of Presentation

A 74-year-old man with ischemic cardiomyopathy was referred for management of recurrent symptomatic VT requiring repeated implantable cardioverter-defibrillator therapies.

## Past Medical History

His cardiac history included previous coronary artery bypass grafting in 1996 and percutaneous coronary intervention in 2013. An implantable cardioverter-defibrillator was inserted in June 2013 for secondary prevention after a cardiac arrest due to VT.

## Investigations

In November 2022, device interrogation revealed multiple episodes of VT despite treatment with sotalol 80 mg twice daily. Sotalol was discontinued, and amiodarone 200 mg daily was initiated. Echocardiography demonstrated a left ventricular (LV) ejection fraction of 30%, with diffuse akinesis and basal inferolateral wall thinning, consistent with scar.

The patient underwent endocardial VT ablation in December 2022 using a contact force–sensing RF catheter and high-density mapping. Substrate mapping in sinus rhythm demonstrated a deceleration zone in the basal inferolateral LV. VT was induced (Right Bundle Left Superior axis transition at V_2_), and activation mapping was performed. Only a partial diastolic pathway was recorded, while the isthmus could not be clearly delineated; predominantly far-field diastolic electrograms were interpreted as suggesting an epicardial extension of the circuit.

Ablation was performed targeting the deceleration zone identified at the basal inferolateral aspect of the LV. This resulted in noninducibility of VT despite aggressive programmed ventricular stimulation. Amiodarone was discontinued after ablation.

In November 2025, the patient presented with recurrent VT episodes, persisting despite the reinitiation of amiodarone, prompting repeat ablation.

## Management

**Figure 1:**
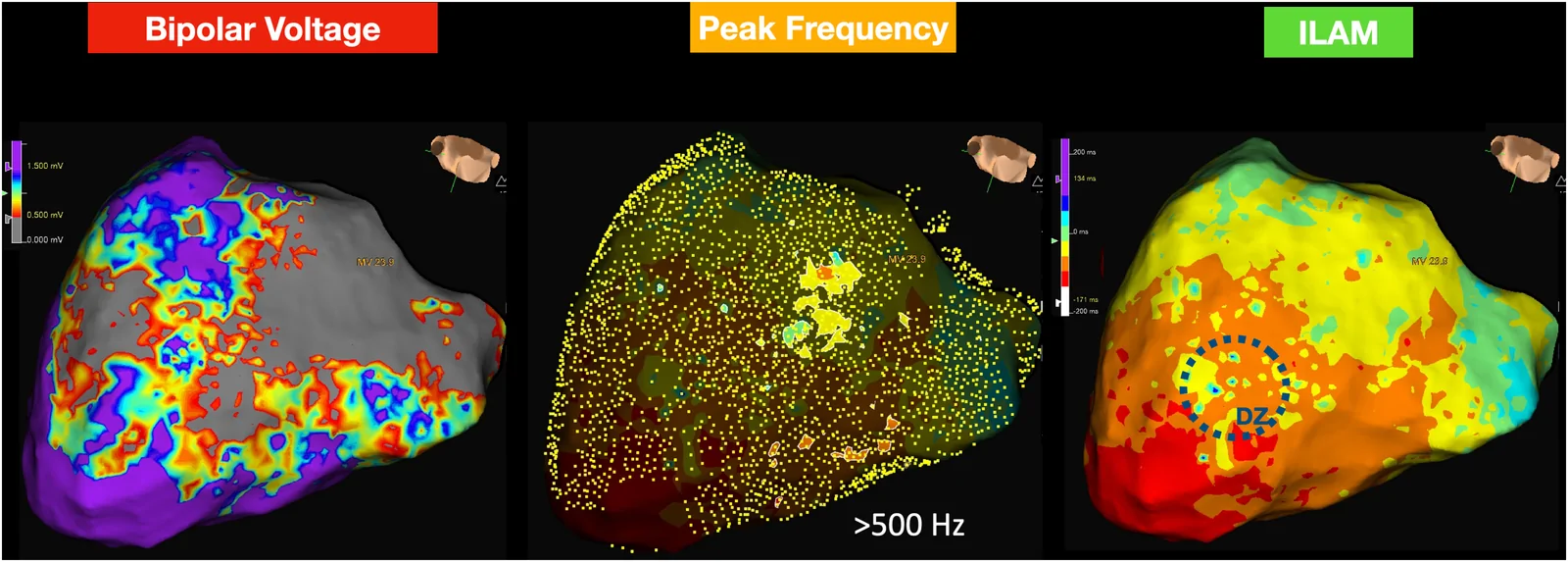
High-Density Electroanatomic Maps of Left Ventricle (Left) High-density bipolar voltage map demonstrating a large scar area of the inferior left ventricle, extending from the base to the apex, sparing the apical tip. (Middle) Peak frequency map, set to >500 Hz, demonstrating a discrete zone of interest along the basal mid-LV inferior wall. (Right) Isochronal late activation map (ILAM) demonstrating a discrete deceleration zone along the mid–left ventricle inferior wall toward the apex.

The patient was brought to the electrophysiology laboratory, and the procedure was performed under general anesthesia. Venous access was obtained via the right femoral vein, and a transseptal approach was used to access the LV. High-density electroanatomic mapping was performed in right ventricular paced rhythm using a multielectrode high-density mapping catheter (Advisor HD Grid X Mapping Catheter, Sensor Enabled, Abbott). High-density bipolar voltage mapping demonstrated a large scar area of the inferior LV, extending from the base to the apex, sparing the apical tip. Isochronal late activation mapping demonstrated a discrete deceleration zone along the mid-LV inferior wall, and a peak frequency map, set to >500 Hz, demonstrated a discrete zone of interest more basally along the LV inferior wall (Figure 1).

**Figure 2:**
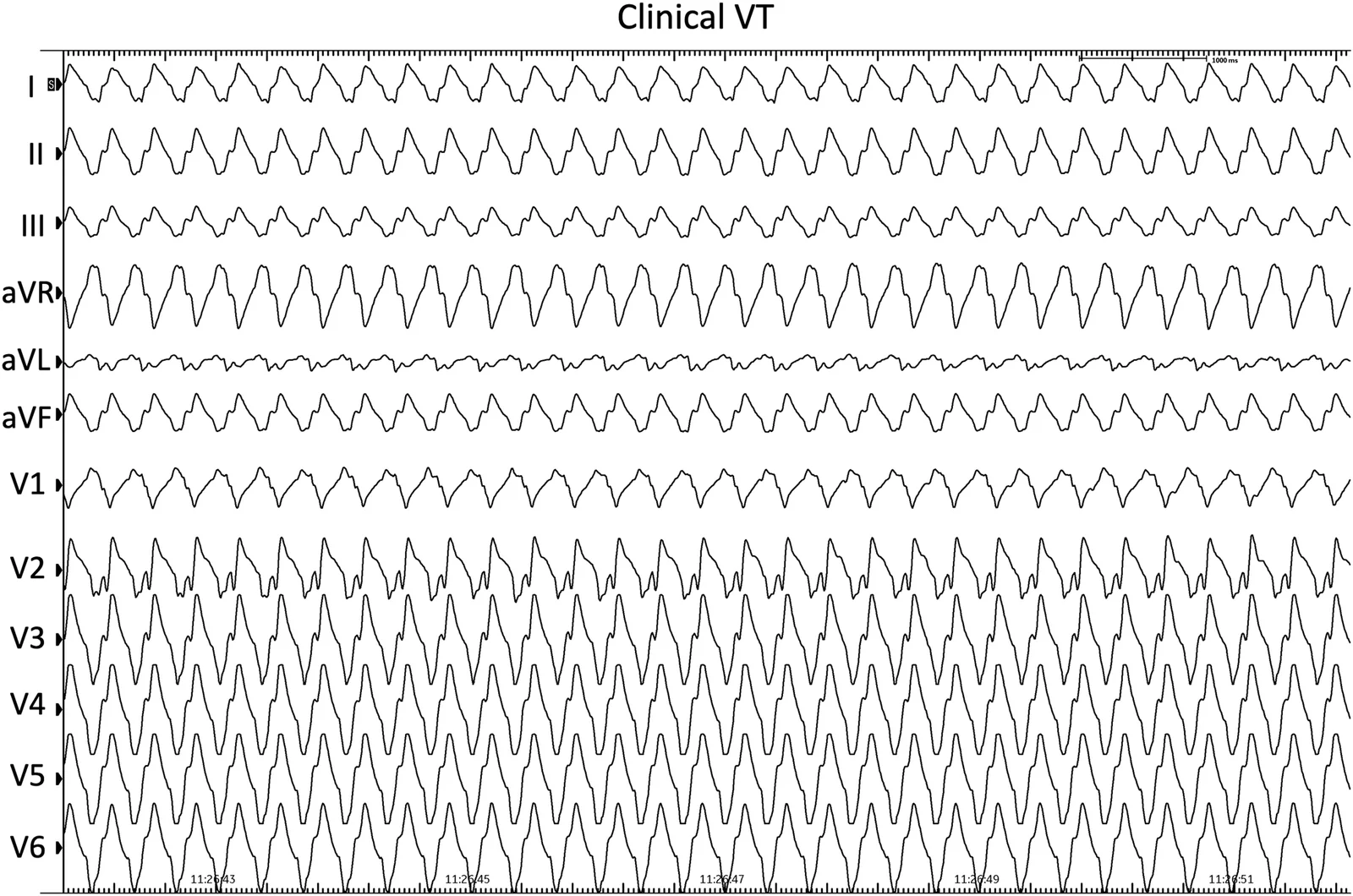
12-Lead Electrocardiogram 12-lead electrocardiogram of the clinical VT demonstrating a right bundle right superior axis with transition at lead V_2_. VT = ventricular tachycardia.

Strategic multielectrode activation mapping of VT was anticipated, given the likelihood of poor hemodynamic tolerance. This was confirmed upon spontaneous VT initiation induced by mechanical premature ventricular complexes. Inotropic/vasopressor support allowed limited activation mapping. VT morphology demonstrated a right bundle right superior axis with transition at lead V_2_ (Figure 2).

**Figure 3:**
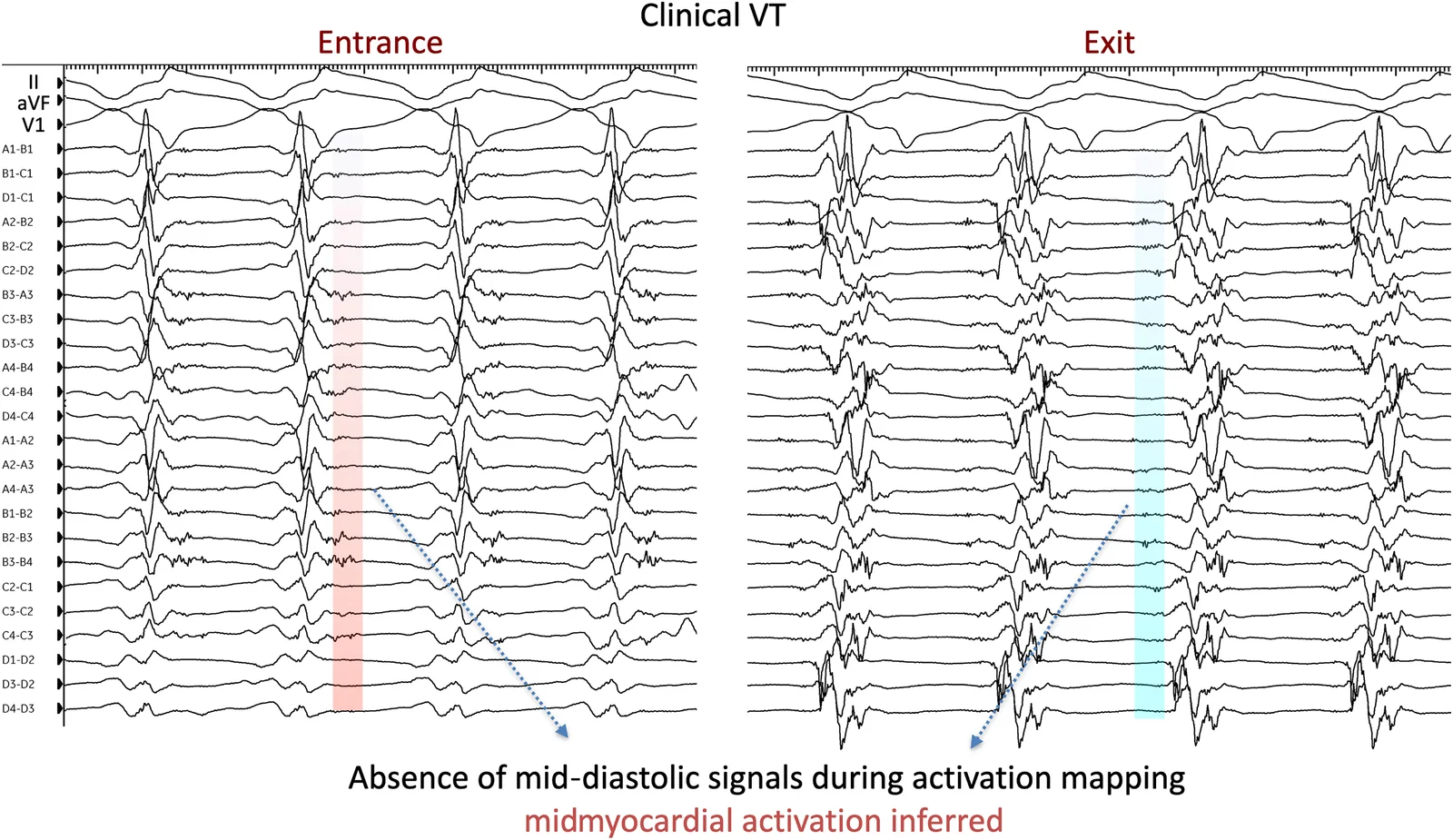
Strategic Multielectrode Activation Mapping of VT Sequential catheter movements on the endocardial surface delineate the diastolic pathway with a clear absence of mid-diastolic electrograms, inferring midmyocardial/epicardial activation. VT = ventricular tachycardia.

**Figure 4:**
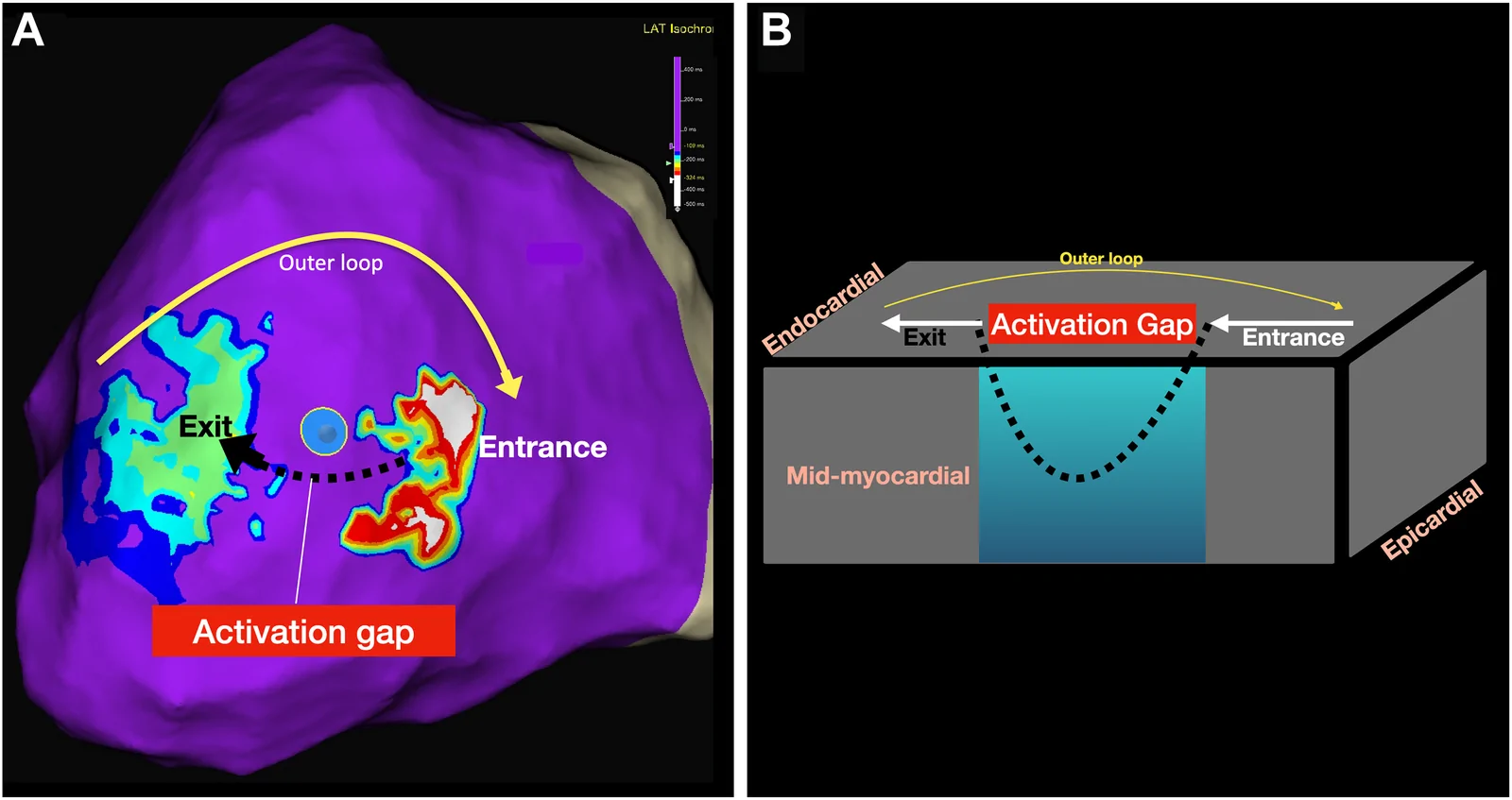
Activation Map of Multiplanar Ventricular Tachycardia (A) Ventricular tachycardia activation map with midmyocardial/epicardial isthmus. The wave of propagation progresses from the entrance on the endocardium, then disappears beneath the surface, manifested as an absence of signals, and finally re-emerges at the exit. (B) Graphical representation of the multiplanar nature of the ventricular tachycardia circuit. The activation gap is in fact the wave of propagation “diving under” the endocardial surface. LAT = local activation time.

Diastolic pathway mapping was performed by sequentially positioning the catheter, beginning from the site of conduction slowing during paced rhythm, and sequentially identifying low-amplitude, long-duration diastolic signals in the first third (0%-35%) of the diastolic interval, corresponding to the entrance; middle third (36%-65%) of the diastolic interval, corresponding to the isthmus; and the last third (66%-100%) of the diastolic interval, corresponding to the exit, as previously described.^4^ VT entrance was recorded along the basal inferolateral LV wall, followed by the disappearance of endocardial diastolic signals and then the reappearance of exit diastolic activity more apically, consistent with a multiplanar circuit (Figure 3). Activation mapping, with the window of interest set at the diastolic phase from QRS offset to QRS onset, demonstrates a clear activation gap at the endocardial surface (Figure 4). Entrainment maneuvers were not pursued because of the patient’s limited hemodynamic stability.

**Figure 5:**
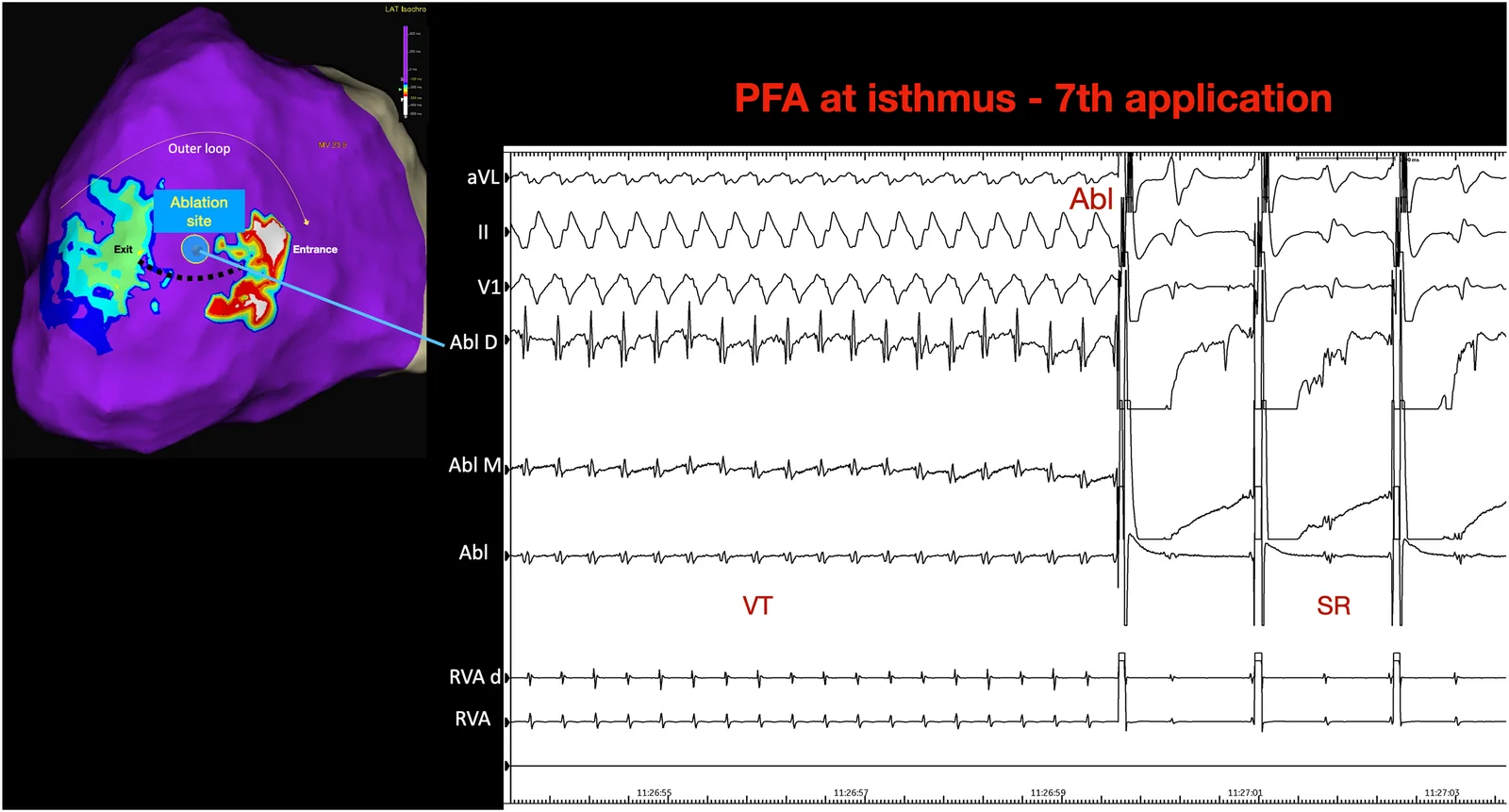
Positioning the Ablation Catheter Endocardially Atop the Suspected Midmyocardial/Epicardial Isthmus VT termination occurred upon the seventh application using an R-wave–gated, 5-burst, 2,200-V waveform ablation catheter. Termination likely occurred upon increasing the ablation depth achieved with successive applications at the same site. Abl = ablation; Abl d = ablation catheter distal; Abl M = ablation catheter mid; PFA = pulsed field ablation; RVA = right ventricular apex proximal; RVA d = right ventricular apex distal; SR = sinus rhythm; VT = ventricular tachycardia; other abbreviations as in Figure 4.

**Figure 6:**
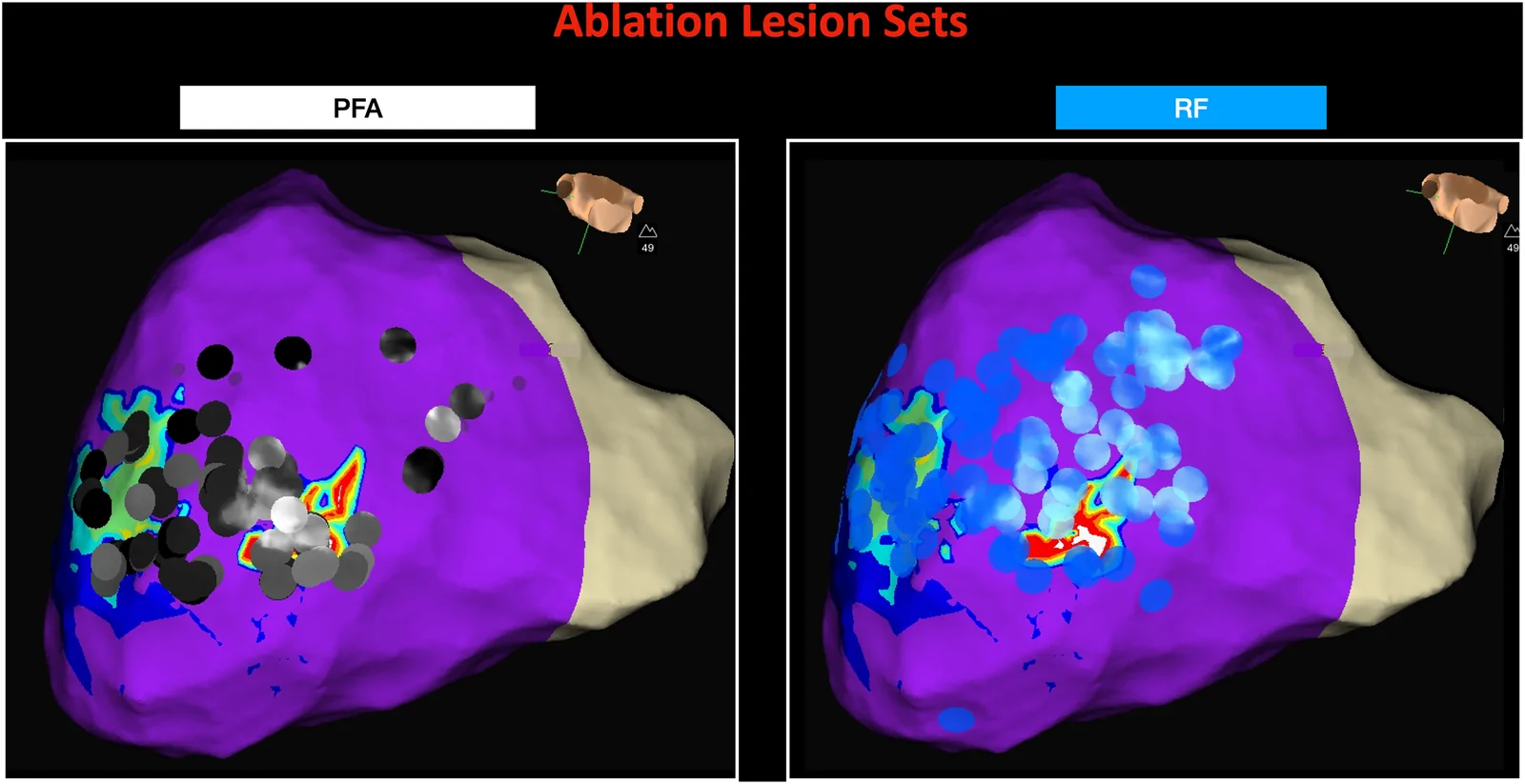
Respective Ablation Lesion Sets of PFA and RFA Overlayed Upon the Ventricular Tachycardia Activation Map Ablation targeted interruption of the re-entrant activation at the isthmus, as well as the funnels at the entry (red) and exit (turquoise and blue) of the ventricular tachycardia channel. PFA = pulsed field ablation; RF = radiofrequency; RFA = radiofrequency ablation.

A dual-energy RF-PF, contact force–sensing, flexible, irrigated-tip catheter (TactiFlex Duo Ablation Catheter, Sensor Enabled, Abbott) was used for ablation. The catheter was positioned directly above the suspected midmyocardial/epicardial isthmus in the absence of near-field diastolic signals. PFA was delivered in a biphasic monopolar configuration at 2,200 V, with 5 R-wave–gated bursts per application and an irrigation flow rate of 13 mL/min. Upon the seventh application of PF stacked at this site, VT terminated (Figure 5). A total of 142 PF applications were applied, targeting the entirety of the diastolic pathway. PF lesion applications were consolidated with RF lesions at 50 W, targeting impedance drops of 10% (Figure 6).

## Outcome and Follow-Up

VT was noninducible despite repeated attempts with programmed extrastimulation after the initial 8 applications at the isthmus site. No St-T wave changes were appreciated during the delivery of PFA in the ventricle, and no signs of coronary vasospasm were observed during the case.

Follow-up at 30 days postablation showed no short-term complications, and device interrogation revealed no VT recurrence.

## Discussion

Percutaneous pericardial access for epicardial VT ablation may not be feasible in patients with previous cardiac surgery because of pericardial adhesions or the increased risk of complications such as graft injury. Furthermore, epicardial access carries a non-negligible risk, with reported complication rates of approximately 5% for major and 2% for minor events in experienced centers^5^; therefore, its indication must be carefully considered.

Nonetheless, epicardial ablation remains an important strategy for targeting complex myocardial scars. Recent cardiac imaging data support the importance of substrate depth definition in determining ablation strategy. In a prospective study using cardiac magnetic resonance imaging, the maximal depth of scar channels was predictive of VT recurrence after endocardial-only ablation. A cutoff of approximately 7 mm was associated with improved outcomes when an exclusively endocardial approach was used. These findings suggest that deeper substrates may require alternative strategies to achieve effective lesion formation and subsequent improved clinical outcomes or reduced VT recurrences.^6^

Lesion depth limitations of RF ablation are well documented. Ischemic scars may limit conductive heat spread, thereby restricting lesion depth and contributing to incomplete substrate modification.^7^

Alternative strategies aimed at increasing lesion depth from an endocardial position have been explored, including multipolar ablation^8^ and ultra-low-temperature cryoablation.^9^

In our case, the absence of mid-diastolic signals during activation mapping across the diastolic corridor infers a midmyocardial/epicardial extension of the circuit, in this specific case, at the level of the isthmus.

Recent data evaluating lesion depth with PFA in ventricular tissue using a flexible irrigated-tip catheter appears promising: in an immediate swine model, ventricular lesions created using an R-wave–gated, 5-burst, 2,200-V waveform ablation catheter can reach median depths of 6.8 mm at 10 bursts (2 applications), with depths increasing with successive applications, reaching 9.1 ± 2.3 mm at 40 bursts at the same site.^10^^,^^11^

The termination of VT in our case, and subsequent noninducibility, occurred not on the first but the seventh consecutive PF application at the site, consistent with the deeper depth penetration that was required. This is in contrast to near-immediate cessation often recorded during single planar VT, with RF transecting the isthmus. The addition of colocalized RF ablation at these sites may have synergistically facilitated deeper penetration contributing to the end-study noninducibility. Nonetheless, optimal pulse configuration targeting ventricular tissue, as well as the order of RF-PF vs PF-RF delivery, has yet to be defined.

## Conclusions

Epicardial access for VT ablation can be challenging, particularly in the setting of previous cardiac surgery. Dual-energy ablation using RF and PF ablation, applied endocardially, may enhance lesion penetration and facilitate modification of midmyocardial/epicardial substrates. In this case, a dual-energy approach enabled successful ablation of a 3-dimensional VT circuit, with arrhythmia termination upon targeted stacked endocardial PF applications at the midmyocardial/epicardial isthmus site. Further studies are needed to better define the role of dual-energy ablation in the management of complex VT substrates.

## Funding Support and Author Disclosures

Dr Hadjis has received consultant fees from Biosense Webster and Abbott. Dr Cerantola has received consultant fees from Medtronic. Dr Marques has reported that she has no relationships relevant to the contents of this paper to disclose.
